# Sleep-Disordered Breathing in an Infant With Achondroplasia and Foramen Magnum Stenosis

**DOI:** 10.7759/cureus.56291

**Published:** 2024-03-16

**Authors:** Claire Feller, Egambaram Senthilvel

**Affiliations:** 1 Pediatrics, School of Medicine, University of Louisville, Louisville, USA

**Keywords:** sleep-disordered breathing, surgical outcome, foramen magnum stenosis, achondroplasia, infant

## Abstract

Sleep-disordered breathing (SDB) is a frequently recognized comorbidity in infants and children with achondroplasia due to alterations in craniofacial and upper airway anatomy. Foramen magnum stenosis and cervicomedullary compression can be associated with SDB in this population, requiring prompt evaluation by multidisciplinary teams. Untreated SDB is associated with adverse cardiovascular, metabolic, and behavioral effects in children, necessitating early screening and treatment of underlying causes. Cervicomedullary compression is also associated with increased mortality and sudden infant death in infants with achondroplasia. Management of SDB in children with achondroplasia may involve a combination of neurosurgical intervention, adenotonsillectomy, and/or continuous positive airway pressure (CPAP). We recognize a need for increased physician awareness of the recommended screening guidelines to optimize health outcomes for children with achondroplasia. In this report, we describe a case of a five-month-old infant with achondroplasia and severe SDB diagnosed by polysomnography and was found to have moderate-to-severe foramen magnum stenosis identified by MRI. Subsequently, this infant underwent foramen magnum decompression, which improved the severe SDB and was followed up for five years. Our case illustrates the importance of early screening in infants with achondroplasia for SDB to prevent further sequelae.

## Introduction

Achondroplasia is the most common skeletal dysplasia, with an estimated birth prevalence of about one in 25,000-30,000 [1]. Achondroplasia occurs due to an autosomal dominant mutation in the fibroblast growth factor receptor 3 (FGFR3) gene that frequently occurs sporadically [1-4]. Children with achondroplasia present features such as short stature with proximally shortened limbs, macrocephaly, frontal bossing, and midface retrusion [1,2]. Foramen magnum stenosis is one of the most severe complications in infants with achondroplasia and can potentially lead to brainstem compression, sleep-disordered breathing (SDB), and even sudden death [5]. Obstructive sleep apnea (OSA) is highly prevalent in children with achondroplasia compared to normal children due to risk factors such as adenotonsillar hypertrophy, midface hypoplasia, foramen magnum stenosis, and obesity [5-8]. Untreated SDB has been associated with adverse neurocognitive, cardiovascular, and metabolic effects in children [9-12]. As the presence of SDB may be associated with cervicomedullary compression in children with achondroplasia, abnormalities on polysomnography (PSG) should prompt neuroimaging and may warrant neurosurgical evaluation based on magnetic resonance imaging (MRI) findings. In this case report, we discuss the evaluation and management of SDB in an infant with achondroplasia and review the current recommendations for early screening and intervention.

## Case presentation

A five-month-old infant with a known history of achondroplasia was referred by genetics for an evaluation in the sleep disorder clinic for concern of SDB. She was born at 34 weeks and was discharged home on day 6 of life on room air. Upon presentation at the sleep clinic, the patient had worsening snoring for the past month, with no episodes of gasping, choking, or apneas. Medical history was also significant for gastroesophageal reflux disease, treated with omeprazole. On exam, the patient presented with features of achondroplasia including frontal bossing, midface hypoplasia, macrocephaly, and shortened limbs. Tonsils were grade 1. A nocturnal PSG (Philips Respironics Alice 3) was ordered to evaluate for SDB. PSG variables are shown in Table 1.

**Table 1:** Pre- and post-operative (three months) and five years of age polysomnographic findings PSG, polysomnogram; TST, total sleep time; REM, rapid eye movement; AHI, apnea-hypopnea index

PSG variables	First PSG	PSG three months post-operatively	PSG at five years of age
Sleep efficiency, %	84.6	85.8	97.4
Sleep onset latency, min	54	59	8.5
Wake after sleep onset, min/TST	20.5	7.8	4.7
REM onset latency, min	43	46	198.5
Stage N1 sleep, % of TST	0.9	0	0.1
Stage N2 sleep, % of TST	47.7	44.3	46.4
Stage N3 sleep, % of TST	20.2	15.7	31.2
Stage REM sleep, % of TST	31.2	40.0	22
Total # of obstructive apneas	43	6	17
Total # of obstructive hypopneas	61	24	19
Total # of central apneas	30	7	1
AHI/hour	19.7	5.5	4.6
Obstructive AHI	15.3	4.5	4.4
Central AHI, events/h TST	4.4	1	0.1
Respiratory arousal index, events/h TST	0	11.8	6.4
Oxygen saturation nadir	74	85	89

As suspected, PSG demonstrated SDB with features of both obstructive and central sleep apnea, with an apnea-hypopnea index (AHI) of 19.7 and central AHI of 4.4 (obstructive apnea-hypopnea indexes (OAHI) of 15.3, O_2_ desaturation nadir of 74% on room air, and no hypoventilation noted). AHI describes the average of both apnea and hypopnea events per hour of sleep. Of note, specific AHI values correlating with mild-to-severe disease are not well defined in pediatrics [13]. Clinically, we use the criteria of 1-5 as mild, 5.1-10 as moderate, and above 10 as severe.

Our case demonstrated a combination of both obstructive and central sleep apnea. We discussed the role of treatment with continuous positive airway pressure (CPAP) versus oxygen supplement therapy and ultimately decided to perform a CPAP titration study (Table 2). At a CPAP setting of 7 cm H_2_O, AHI normalized to 0.9, and O_2_ desaturation nadir improved to 93%.

**Table 2:** CPAP titration result table EPAP, expiratory positive airway pressure; REM, rapid eye movement; NREM, non-rapid eye movement; AHI, apnea-hypopnea index; OSH, obstructive hypopnea; OSA, obstructive sleep apnea; CSA, central sleep apnea; PLM, periodic limb movement; SaO_2_, oxygen saturation; CPAP, continuous positive airway pressure

EPAP	Total min	Sleep min	REM min	NREM min	Supine min	Total AHI	OSH (/hr)	OSA (/hr)	CSA (/hr)	Arousal (/hr)	PLM (/hr)	SaO_2_ nadir	SaO_2 m_ean
6	55.2	38.2	0.0	38.2	38.2	0.0	0.0	0.0	0.0	1.6	0.0	97	98
7	150.4	138.4	31.9	106.5	138.4	0.9	0.0	0.0	0.9	4.3	0.0	93	98
8	111.4	110.9	20.4	90.5	110.9	3.2	0.0	0.0	3.2	7.6	0.0	92	99
9	69.3	66.8	30.8	36.0	66.8	5.4	0.9	0.0	4.5	9.9	0.0	88	99
10	46.9	46.9	7.9	39.0	46.9	1.3	0.0	0.0	1.3	2.6	0.0	95	99

Thus, the patient was started on CPAP therapy with a nasal mask and tolerated it well. We also referred the patient to an otolaryngologist for upper airway evaluation, which was normal. Magnetic resonance imaging (MRI) and computed tomography (CT) imaging were arranged to rule out foramen magnum stenosis due to the combination of obstructive and central events observed in this patient. MRI is also useful for demonstrating edema, hydrocephalus as well as cerebrospinal fluid (CSF) flow limitation caused by stenosis.

**Figure 1:**
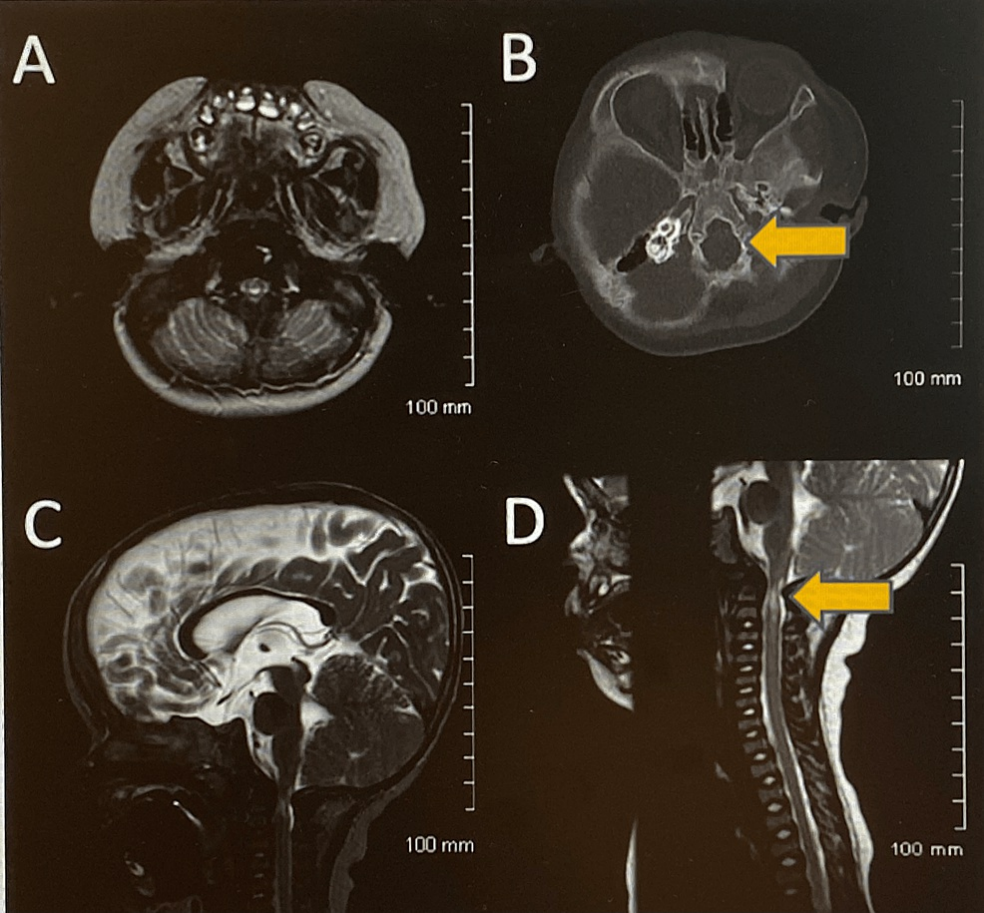
MRI and CT head and neck findings Images A (axial T2 MRI), C (sagittal T2 MRI), D (sagittal T2 MRI) - stigmata of achondroplasia with moderate-to-severe foramen magnum narrowing with kinking of the cervicomedullary junction with patchy T2 hyperintense signal abnormality in the upper cervical spinal cord and cervicomedullary junction, arrow in the sagittal image D. Image B (non-contrast CT) - small skull base with narrowing of the foramen magnum, arrow in the image. MRI, magnetic resonance imaging; CT, computed tomography

**Figure 2:**
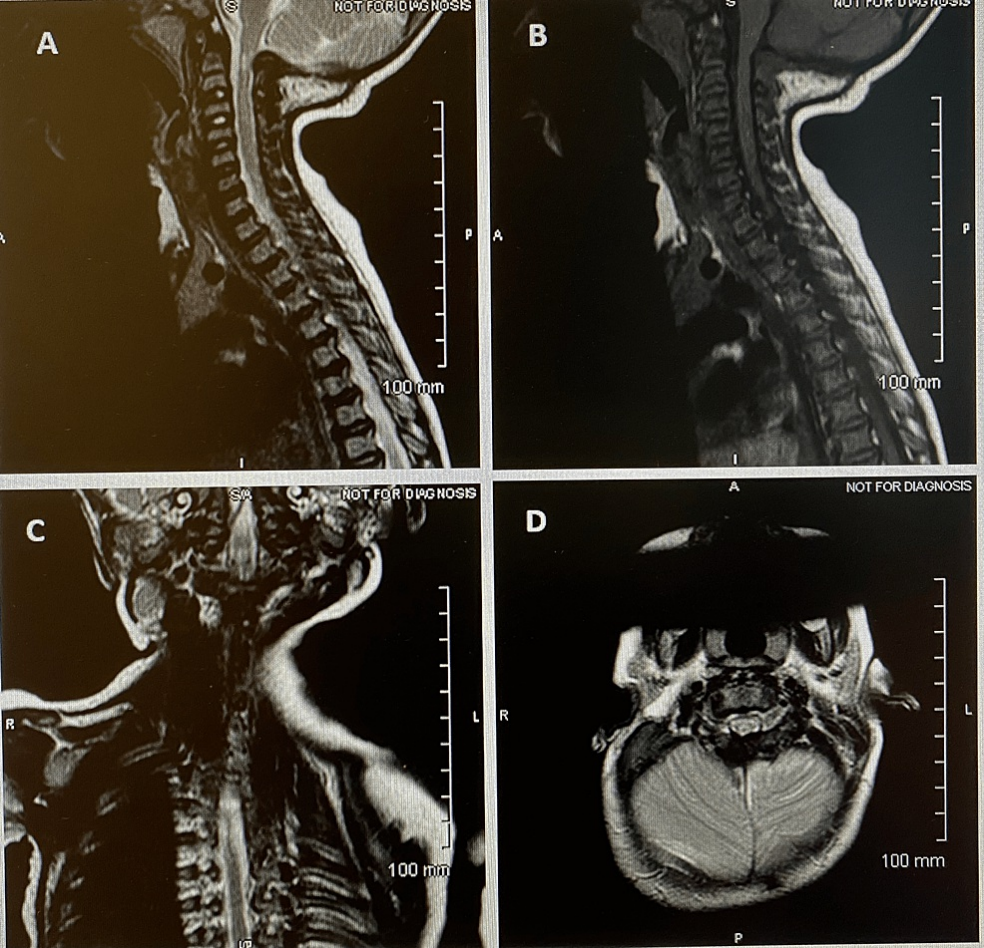
Post-operative MRI images Images A (sagittal T2), B (sagittal T1), C (coronal T2), and D (axial T2) - MRI images from 2017 February post-foramen magnum decompression surgery - improvement in foramen magnum stenosis with increased CSF space ventral to the cervicomedullary junction, axial and sagittal images. MRI, magnetic resonance imaging; CSF, cerebrospinal fluid

The MRI (Figure 1) showed moderate-to-severe foramen magnum narrowing with kinking of the cervicomedullary junction; patchy T2 hypersensitivity abnormalities in the cervical cord and cervicomedullary junction were noted. The patient was subsequently hospitalized for foramen magnum decompression without duroplasty. Post-operatively, the patient’s caregiver reported significant improvement in the patient’s snoring episodes while sleeping. A follow-up PSG was performed three months after surgery (Table 1), which showed an AHI of 5.5 with an O_2_ saturation nadir of 85%. Wake after sleep onset reduced, arousal index increased, and total rapid eye movement (REM) sleep time improved compared to the baseline study in addition to respiratory parameters. Follow-up MRI showed significant improvement as in Figure 2. The patient continued to do well and no longer required CPAP at this time. At five years of age, a follow-up PSG (Table 1) showed an AHI of 5 and did not require any further intervention for mild sleep apnea at that time. 

## Discussion

Both central and OSA are well-described comorbidities of achondroplasia, affecting over half of infants with achondroplasia and frequently continuing into adulthood [8]. It is thought that the pathophysiology of SDB in patients with achondroplasia relates to upper airway crowding as well as compression of the brainstem in the setting of a stenotic foramen magnum, as OSA has been seen to improve in these patients after posterior fossa decompressive operations [14]. If left untreated, OSA can contribute to neurocognitive dysfunction in children in a dose-dependent manner, negatively impacting domains such as attention and memory [9]. A study by Csábi et al. found that children with SDB had significantly higher attention-deficit/hyperactivity disorder (ADHD) scale ratings compared to age- and gender-matched controls, noting that even mild degrees of SDB are associated with emotional and behavioral difficulties in children [10]. Pediatric OSA also increases the risk of adverse cardiovascular and metabolic effects. Moderate-to-severe OSA in children is associated with elevated blood pressure, with hypertension often persisting into adulthood in these patients if left untreated [11]. Furthermore, OSA is a recognized risk factor for dyslipidemia in both obese and non-obese children [12]. In obese children, moderate and severe OSA is associated with alanine transaminase (ALT) elevation, suggesting that the degree of OSA may be associated with non-alcoholic fatty liver disease (NAFLD) [12]. Thus, it is essential to recognize and treat all cases of OSA in children to ensure optimal growth, cardiovascular, neurocognitive, and metabolic outcomes.

Central sleep apnea has been observed in children with congenital malformations affecting the skull base and foramen magnum, such as achondroplasia, Chiari malformation, and Joubert syndrome [15]. The etiology of central sleep apnea in achondroplasia remains somewhat elusive and is not fully understood, but some have hypothesized that it is associated with compression of the brainstem and subsequent dysfunction of central respiratory control centers [16]. Additionally, though no clear mechanism for sudden infant death exists for patients with achondroplasia, some theorize that it may be related to the increased prevalence of apneic events during sleep coupled with a diminished arousal response [17].

Due to the high prevalence of SDB in achondroplasia, The American Academy of Pediatrics (AAP) recommends PSG for all infants with achondroplasia within the first month of life [2]. This recommendation remains the best practice since the previous AAP guidelines published in 2005, which recognized the need for early evaluation by PSG in this population [18]. In the case outlined above, the abnormal PSG findings demonstrated both obstructive and central events and prompted neuroimaging, which showed moderate-to-severe foramen magnum stenosis. As cervicomedullary compression in infants with achondroplasia may be associated with an increased risk for sudden infant death as well as central sleep apnea, it thus warrants surgical intervention [19]. If cervicomedullary compression is present, decompressive surgery is recommended for patients with achondroplasia, ideally before the age of four years and prior to symptom development [20]. PSG is an essential screening tool for identifying apparently asymptomatic patients, as it may reveal SDB and prompt further evaluation to assess for cervicomedullary compression and associated foramen magnum stenosis [20].

Because of the risk of cervical spinal cord compression in this population, the AAP recommends that neuroimaging be performed in combination with PSG for all infants with achondroplasia in the first month of life [2]. Despite these guidelines, many infants and children with achondroplasia fail to receive adequate screening. In their 2019 study, Nadel et al. found that within a cohort of 236 children with achondroplasia, only 13.9% received both PSG and neuroimaging screening in accordance with the AAP’s 2005 recommendations. While screening rates improved after these guidelines were published, from 32.9% in children born between 2001 and 2007 to 60.3% in children born from 2008 to 2014, a significant screening gap remains, leaving infants at risk of complications from sleep apnea as well as cervicomedullary compression.

Because of the high prevalence of OSA in infants and children with achondroplasia, routine otolaryngology evaluation is also recommended, as adenoidectomy and/or tonsillectomy are associated with decreased OSA in this population. Of note, adenotonsillar hypertrophy is a significant risk factor for the development of pediatric OSA in otherwise healthy children, for which adenotonsillectomy is recommended by the AAP [13]. Repeat PSG is recommended after surgical intervention to gauge the degree of OSA improvement. As noted by Booth et al., management of OSA in children with achondroplasia is often difficult, as some degree of OSA often persists in these patients even after multiple surgical interventions. In their retrospective study of children with achondroplasia and OSA, they found that while 72.7% of children demonstrated an improvement in OAHI after receiving upper airway surgery, only 18.2% experienced full OSA resolution post-operatively. If patients continue to show signs of OSA after upper airway surgery, continuous CPAP may be warranted, along with regular follow-up with sleep medicine to monitor their OSA severity over time [4]. In the present case, the patient’s SDB improved post-neurosurgical decompression, and they no longer required CPAP therapy post-operatively.

## Conclusions

This case of a five-month-old infant with achondroplasia presenting with SDB and followed up for five years illustrates the importance of early screening with PSG and neuroimaging and prompt intervention to reduce the potential for adverse outcomes. Early PSG is essential to identifying potentially severe causes of SDB in this population, as is neuroimaging if PSG demonstrates abnormalities. Given the risk of craniocervical junction abnormalities in this population and their associated morbidity and mortality, prompt evaluation with the involvement of neurosurgical intervention can improve outcomes for these patients. This case study may increase provider awareness of the existing screening guidelines for children with achondroplasia to ensure that all children receive prompt and potentially lifesaving interventions.
